# Keratinocytes Migration Promotion, Proliferation Induction, and Free Radical Injury Prevention by 3-Hydroxytirosol

**DOI:** 10.3390/ijms22052438

**Published:** 2021-02-28

**Authors:** Mario Abate, Marianna Citro, Simona Pisanti, Mariella Caputo, Rosanna Martinelli

**Affiliations:** Department of Medicine, Surgery and Dentistry “Scuola Medica Salernitana”, University of Salerno, Via Salvador Allende, Baronissi, 84081 Salerno, Italy; mabate@unisa.it (M.A.); mcitro@unisa.it (M.C.); spisanti@unisa.it (S.P.); macaputo@unisa.it (M.C.)

**Keywords:** 3-hydroxytyrosol, wound healing, oxidative stress, cosmeceuticals

## Abstract

3-hydroxytyrosol (HT) is the main phenolic compound found in olive oil with known antioxidant, anti-inflammatory, and antimicrobial properties in several dermatological conditions, both when taken in the form of olive oil or pure in cosmeceutical formulations. To date, its direct effect on the wound healing process and the molecular mechanisms involved have not yet been elucidated. Thus, in the present study, we aimed to explore its effects in vitro in epidermal keratinocyte cultures focusing on the molecular mechanism implied. HT was able to induce keratinocyte proliferation in the low micromolar range, increasing the expression of cyclin dependent kinases fundamental for cell cycle progression such as CDK2 and CDK6. Furthermore, it increased cell migration through the activation of tissue remodeling factors such as matrix metalloproteinase-9 (MMP-9) protein. Then, we evaluated whether HT also showed antioxidant activity at this concentration range, protecting from H_2_O_2_-induced cytotoxicity. The HT prevented the activation of ATM serine/threonine kinase (ATM), Checkpoint kinase 1 (Chk1), Checkpoint kinase 2 (Chk2), and p53, reducing the number of apoptotic cells. Our study highlighted novel pharmacological properties of HT, providing the first evidence of its capability to induce keratinocyte migration and proliferation required for healing processes and re-epithelialization.

## 1. Introduction

Many beneficial properties have been attributed to the Mediterranean diet (MD) which, to date, represents the best diet for the prevention of numerous diseases. It is indeed well recognized that adherence to the MD is associated with greater longevity thanks to its cardioprotective and general anti-inflammatory properties and with a lower incidence of chronic degenerative diseases and cancer [1,2,3,4,5].

Over the years, scientific research has tried to identify which molecules enriched in foods that characterize MD pattern such as olive oil, vegetables, fruit, legumes, cereals, fish, and so on, are responsible for its beneficial effects [6,7,8]. At the same time, the identification of these bioactive molecules has increasingly attracted pharmaceutical companies toward the creation of nutraceuticals to be used in the prevention and treatment of numerous diseases.

To date, scientific research has shown that the polyphenols contained in olive oil, the main source of fat at the base of MD, have excellent pharmacological-nutritional properties and it is also thanks to these molecules that MD is so beneficial to health [9,10,11,12].

The main phenolic compound of olive oil is 3,4-dihydroxyphenylethanol (3,4-DHPEA), also known as 3-hydroxytyrosol (HT), which derives from the spontaneous chemical hydrolysis of oleuropein during olives ripening [13,14]. HT is a molecule of high interest for the pharmaceutical industry thanks to its strong antioxidant, anti-inflammatory, antimicrobial, cardioprotective, and neuroprotective properties [15,16,17]. In addition, the continuous research conducted in this field has also shown that this molecule has no undesirable effects even at high doses [18,19], so much so that the EFSA (European Food Safety Authority) recommends a daily intake of at least 5 mg of 3-hydroxytyrosol through extra virgin olive oil use at meals [20,21].

Historically, beyond its nutritional use, olive oil has been employed, along or as an alternative to other plant oils, topically applied on the skin for the treatment of numerous dermatological conditions. Its beneficial properties, beyond the moisturizing action, are due to the antioxidant and anti-inflammatory effects of its constituents such as phenolic compounds and tocopherols. Currently, the prevention and treatment of dermatological diseases and the protection of skin are assuming great importance due to exposure to sunlight without protective factors, pollution, or aging factors. Therefore, given the multiple beneficial activities of olive oil and HT in different physiological and pathological contexts, more and more studies are aimed at evaluating the benefits of such natural molecules [22,23,24].

To date, various scientific studies have shown beneficial properties on the skin of both olive oil and HT, derived from daily oral intake or after topical application. Owen et al. hypothesized and evaluated how dietary intake of polyphenols present in olive oil is associated with a reduction in the incidence of skin cancers [25]. Recent studies conducted in vivo on mice have shown that the daily oral intake of olive oil improves the healing of pressure ulcers, thanks to its anti-inflammatory properties, reducing oxidative damage and promoting dermal reconstruction, which are added to the reduction of nitric oxide synthesis and contrast of reactive oxygen species (ROS) [26]. Furthermore, oral administration of olive oil has been shown to accelerate collagen deposition, myofibroblastic differentiation, and wound contraction [26].

In addition to oral administration, many studies are evaluating the beneficial effects of olive oil on the skin when used in cosmetic formulations. Recent research shows that olive oil creams are effective for treating patients with chronic wounds, having the ability to reduce the size, depth, and edges of the wound, along with the associated pain [27]. Better wound healing has also been associated with the antibacterial effects of polyphenols present in the oil [28], configuring it as also useful in the treatment of burns [28,29]. Some studies suggest that the positive effects of olive oil in promoting wound healing are due to the modulation of early stages of the reparative process such as inflammation and stimulation of dermal reconstruction [30].

However, in addition to testing olive oil as a whole, other studies have been aimed to investigate the properties of its main components [14]. It has been shown that HT has great antioxidant properties, even higher than vitamin E, with remarkable radical-scavenging effects in vitro, responsible for the cytoprotective activity [22].

However, HT is extensively metabolized after oral administration, which leads to the formulation of HT in a topical vehicle to prolong its pharmacological action and at the same time provide a localized effect [31].

Thanks to these strong antioxidant activities, many pharmaceutical companies are currently trying to create cosmetic formulations based on HT-based antioxidant surfactant emulsions [32].

Recent studies have shown that HT may be a potential alternative therapeutic agent for the treatment of rheumatoid arthritis, in fact, it mitigates hydrocortisone systemic adverse effects when co-administered [33]. HT provides additional anti-inflammatory and antioxidant benefits in the treatment of atopic dermatitis and shows antipsoriasic-beneficial properties [18,34,35].

Despite HT action against aging or dermal damage, its direct effect on wound healing and the molecular mechanisms involved have not yet been elucidated.

To date, its wide range of biological properties has mainly been attributed to its strong antioxidant activity [36], but it is increasingly clear that so many biological activities observed in vivo cannot be traced back to antioxidant and anti-inflammatory activity alone.

In this study we analyzed the molecular mechanisms underlying the properties of HT using the human keratinocytes cell line HaCaT, a well characterized cell line with a wide use as an in vitro model of epidermal damage.

Our results demonstrate that HT is able to stimulate keratinocyte proliferation and migration by the activation of tissue remodeling factors, in addition to its free radical fighting activity, thus preventing skin aging.

## 2. Results

### 2.1. Evaluation of 3-Hydroxytyrosol (HT) Effect on Human Keratinocytes

**Figure 1:**
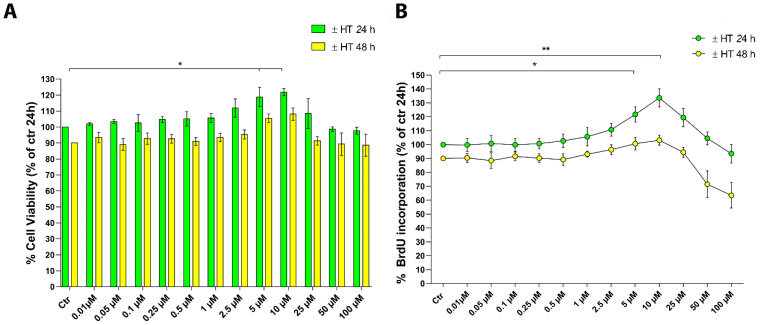
Evaluation of 3-hydroxytyrosol (HT) effect in HaCaT keratinocytes. Cells were cultured in the presence of the indicated HT concentrations (0–100 µM) for 24 or 48 h before the MTT assay (**A**) or BrdU incorporation (**B**). Results are expressed as mean (± SD) and are representative of four independent experiments carried out in triplicate. Data are reported as percentage vs. control (untreated cells at 24 h or 48 h) (2-way ANOVA, * *p* < 0.05, ** *p* < 0.01 vs. control).

The HaCaT cells were cultured with increasing concentrations of HT (0–100 µM) for 24 and 48 h and their cell viability was evaluated by the MTT (3-(4, 5-dimethylthiazolyl-2)-2, 5-diphenyltetrazolium bromide) assay. The results reported in Figure 1A show that HT has no influence on cell viability even at the highest doses. Interestingly, we identified an interval of concentrations (5 and 10 µM) where HT induced a little, but significant increase of cell viability at 24 h (* *p* < 0.05). Therefore, we evaluated whether the increase in cell viability was attributable to an increased number of cells, due to proliferation. HaCaT cells were cultured for the same times and with the same concentrations of HT and cell proliferation was assessed by BrdU incorporation (Figure 1B). HT 5 and 10 µM induced an increase in DNA synthesis only at 24 h, thus effectively stimulating cell proliferation (* *p* < 0.05 and ** *p* < 0.01) whereas at a longer time (48 h), the effect was not so marked, probably due to a depletion of nutrients in the culture medium.

We then decided to perform all subsequent experiments in the 1–10 µM range of concentrations and within 24 h.

### 2.2. HT Induces Expression of Cell Cycle Control Proteins

**Figure 2:**
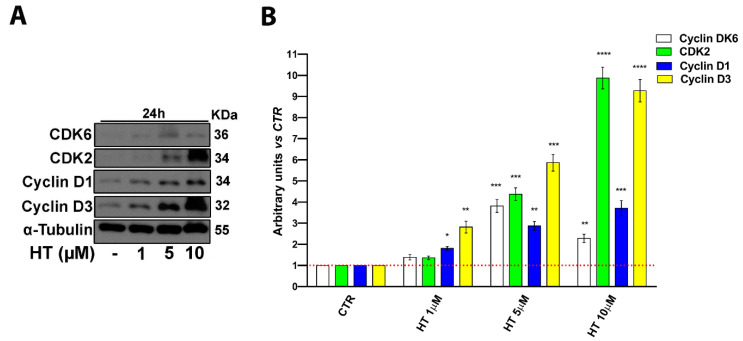
HT induced expression of cell cycle progression proteins. (**A**) Western blot analysis of cyclin D1, CDK6, CDK2, and cyclin D3 in whole cell extracts from HaCaT cells cultured for 24 h with HT 1–10 µM. α-Tubulin was used as a control for protein loading. The panel shows a representative western blot of three different experiments with similar results. (**B**) Histograms represent mean (±SD) in densitometry units of scanned immunoblots from three different experiments (2-way ANOVA, * *p* < 0.05, ** *p* < 0.01, *** *p* < 0.001, and **** *p* < 0.0001).

We next investigated the influence of HT on cell cycle progression, focusing on the molecular pathways through which it carries out the observed biological effects. To this end, we determined by western blot analysis the status of the same proteins specifically involved in cell cycle progression (Figure 2). We treated cells with HT at the most effective doses, 5 and 10 µM for 24 h. According to the proliferation assay results, we observed that HT increased the expression of proteins involved in cell cycle transition from G1 to the S phase such as cyclin D1, D3, Cyclin Dependent Kinase 2 (CDK2), and Cyclin Dependent Kinase 6 (CDK6) (Figure 2A,B).

### 2.3. HT Improves the Migratory Capacity of HaCaT Keratinocytes

**Figure 3:**
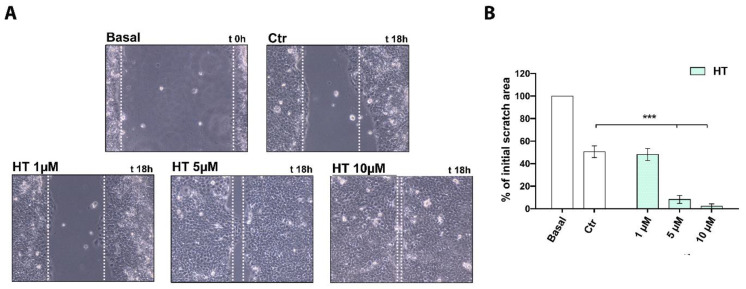
Improvement of the migratory capacity of HaCaT cells exposed to HT. (**A**) Wound healing assay was carried out in HaCaT cells treated for 18 h with HT (1–10 µM) in complete medium. Light microscope images shown are representative of four independent experiments. Dotted white lines indicate the wounded area from the initial scratch. Magnification ×200; (**B**) Bar columns correspond to the mean scratch area expressed as percentage respect to basal time point area. The measurement was carried out in four different experiments. Results are shown as mean (± SD) (2-way ANOVA, *** *p* < 0.001).

Keratinocyte cell migration is essential for the re-epithelialization both after superficial and deeper skin injury such as ulcers [37,38]. For this reason, in order to assess a potential effect of HT on the migratory function of HaCaT cells, we performed a scratch wound assay. The closure of the wounded area, carried out on a monolayer of confluent cells, was monitored over the time. After 18 h of treatment with HT, we observed an enhancement of wound healing at 5 and 10 µM (*** *p* < 0.001) with respect to the control and to a smaller concentration of HT (1 µM), as shown in Figure 3A. Indeed, the wound area, measured as the percentage of initial scratch at time 0, was almost completely closed after 18 h by HT at 5 and 10 µM with respect to untreated control (Figure 3B).

### 2.4. HT Induces Migration-Linked Proteins Expression 

**Figure 4:**
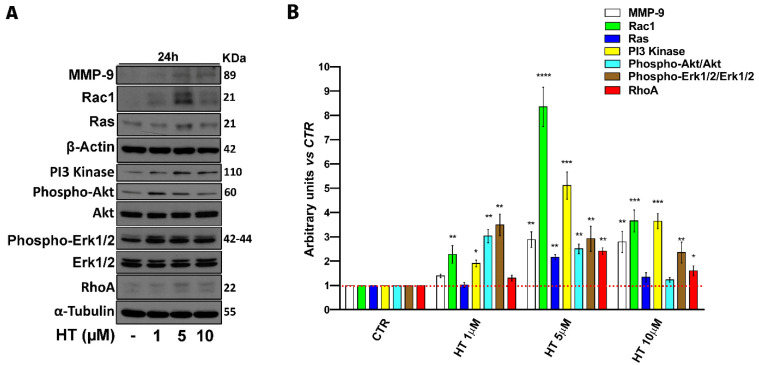
HT induces migration protein expression in HaCaT cells. (**A**) Western blot analysis of MMP-9, Phospho Erk1/2, Erk1/2, Phospho-Akt, Akt, PI3 Kinase, RhoA, Rac1, and Ras in whole cell extracts from HaCaT cells treated for 24 h with HT 1–10 µM. β-Actin and α-Tubulin were used as the control of protein loading. The panel shows a representative western blot of four different experiments with similar results. (**B**) Histograms represent mean (± SD) in densitometry units of scanned immunoblots from four different experiments (2-way ANOVA, * *p* < 0.05, ** *p* < 0.01, *** *p* < 0.001, and **** *p* < 0.0001).

Regulation of epithelial cell migration involves several factors and for this process the activation of cytoskeletal remodeling signaling pathways is essential [38,39,40,41,42,43]. In order to establish the mechanism of HT action on the migration stimulation, we determined the expression of crucial proteins involved in the migration process by western blot. We treated cells with HT at concentrations of 1–10 µM for 24 h. We observed an upregulated expression of proteins that are implicated in cell adhesion, cytoskeletal dynamics, and migration such as extracellular regulated protein kinases (ERK), phosphatidylinositol 3-kinase (PI3 Kinase), protein kinase B (Akt), ras-related C3 botulinum toxin substrate 1 (Rac1), ras homolog family member A (RhoA), GTPase (Ras), but also the activation of matrix metalloproteinase-9 (MMP-9) required for the degradation of the extracellular matrix (Figure 4A,B). These data suggest that exposure to HT provided a driving force in human keratinocyte migration.

### 2.5. HT Contrasts Cytotoxicity and Apoptosis Induced by H_2_O_2_

**Figure 5:**
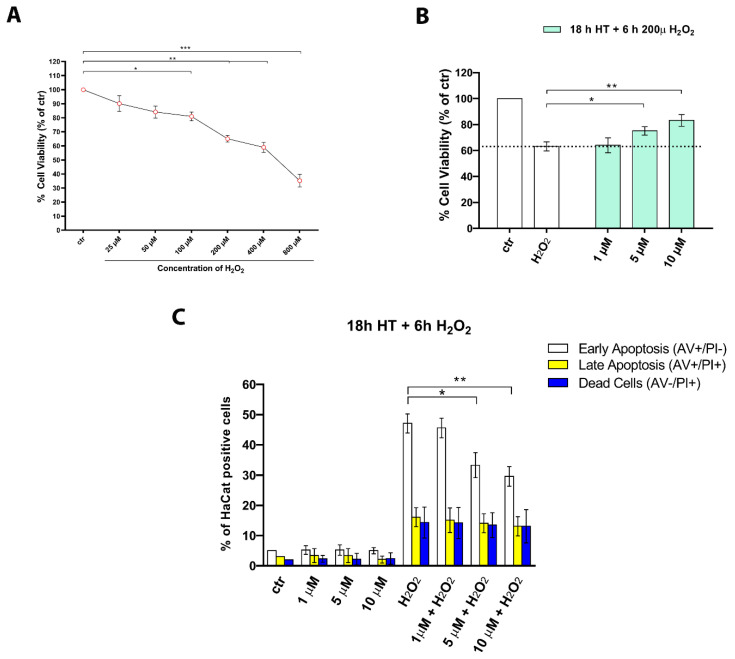
HT ameliorated cytotoxicity and apoptosis induced by H_2_O_2_ in keratinocytes. (**A**) HaCaT cells were cultured for 6 h in the presence of H_2_O_2_ (25–800 µM) before the MTT assay. The results are expressed as means ± SD of three independent experiments and reported as percentage vs. the untreated control (2-way ANOVA, * *p* < 0.05, ** *p* < 0.01, and *** *p* < 0.001). (**B**) HaCaT cells were cultured for 18 h in the presence of HT (1–10 µM) before treatment with H_2_O_2_ for 6 h. The results are expressed as means ± SD of three independent experiments reported as percentage vs. the untreated control (2-way ANOVA, * *p* < 0.05 and ** *p* < 0.01 vs. control). (**C**) Flow cytometric analysis of annexin V and propidium iodide (PI) double staining in HT-pretreated HaCaT cells for 18 h after H_2_O_2_ exposure for 6 h. Bars indicate the total percentage of early (annexin V-positive cells/PI-negative cells) and late apoptotic events (annexin V/PI-double positive cells) as well as necrotic cells (annexin V-negative cells/PI-positive cells). The results are representative of three independent experiments and expressed as mean ± SD (ANOVA, * *p* < 0.05 and ** *p* < 0.01).

To evaluate whether the HT concentrations identified in previous experiments (1–10 µM) could also show antioxidant activity in our experimental model, as previously reported in other cell lines for HT, we tested its effects against H_2_O_2_-induced cytotoxicity and apoptosis. HaCaT cells were exposed to H_2_O_2_ (0–800 µM) for 24 h, and the MTT assay was used as an indicator of cell viability (Figure 5A). We observed a dose-dependent decrease in cell viability following H_2_O_2_ treatment. Cell viability reduction was statistically significant already at 100 µM H_2_O_2_, whereas it was reduced to 65.1% at 200 µM H_2_O_2_ (Figure 5A). It is recognized that concentrations of H_2_O_2_ within 250 µM caused apoptotic death, while higher doses, up to 1000 µM of H_2_O_2_, resulted in an increase in overall cell death [44]. For this reason, we used 200 µM H_2_O_2_ in the subsequent experiments. We observed that 18 h pretreatment of HaCaT keratinocytes with HT at 5 and 10 µM significantly protected the cells from H_2_O_2_-induced cytotoxicity, improving cell viability. After exposure to 200 µM H_2_O_2_ for 6 h, cell viability dropped to 65.1% ± 5.7%, whereas it increased to 75.2% ± 3.3% and 83.2% ± 4.5% by pre-treatment with HT at 5 and 10 µM, respectively (Figure 5B). Subsequently, we performed a cell death analysis by annexin-V and propidium iodide double staining to corroborate the data obtained by the MTT assay. HT pretreatment before H_2_O_2_ exposure resulted in a significant reduction of apoptosis, in particular, inhibiting the percentage of early double positive apoptotic cells (Figure 5C).

### 2.6. HT Prevents Activation of Key DNA Damage-Associated Proteins 

**Figure 6:**
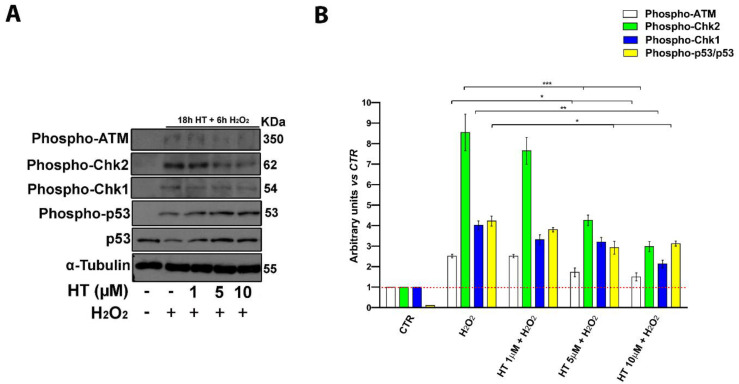
HT prevented the activation of cell death molecular pathways. (**A**) Western blot analysis of Phospho-ATM, Phospho-Chk1, Phospho-Chk2, and p53 (total and phosphorylated) in whole cell extracts from HaCaTs for 18 h in the presence of HT (1–10 µM) and H_2_O_2_ (200 µM) for 6 h. α-Tubulin was used as a control for protein loading. The panel shows a representative western blot of three different experiments performed with similar results. (**B**) Histograms represent mean (±SD) in densitometry units of scanned immunoblots of three different experiments (2-way ANOVA, * *p* < 0.05, ** *p* < 0.01, and *** *p* < 0.001).

ATM serine/threonine kinase (ATM), Checkpoint kinase 1 (Chk1), Checkpoint kinase 2 (Chk2), and p53 are known to play a pivotal role in the control of cell response to external damages, indeed, they are induced by free radicals and involved in apoptosis activation following DNA damage [45,46,47,48]. In order to elucidate the molecular pathways modulated by HT, using H_2_O_2_ to mimic oxidative stress-induced injury (OSI) within a short period, we performed a western blot analysis of ATM, Chk1, Chk2, and p53 proteins (Figure 6). We evaluated the phosphorylation status of these proteins in 18 h pretreated keratinocytes with HT at 1–10 µM, following H_2_O_2_ exposure (Figure 6).

Phospho-ATM, Phospho-Chk1, Phospho-Chk2, and p53 levels were increased by H_2_O_2_-treatment whereas HT treatment significantly reversed these effects at higher concentrations (5 and 10 µM) and the activation of these DNA damage signaling pathways (Figure 6A,B). The results showed that HT protects keratinocytes from the activation of the oxidative stress-induced DNA-damage pathway and subsequent apoptosis activation. 

## 3. Discussion

HT, the most represented polyphenol contained in olive oil, has a strong biological relevance thanks to its antioxidant, anti-atherogenic, anti-inflammatory, neuroprotective, and antitumor activities, which have been demonstrated by accurate scientific studies both in vitro and in animal models, supporting its preventive and pharmacological potential [1,2,3,4,5]. With regard to dermatological diseases, recent studies have shown that the daily consumption of olive oil through the Mediterranean dietary pattern, is associated with a lower incidence of skin tumors, but also with a better healing of pressure ulcers, thanks to a reduction in oxidative damage, a better dermal reconstruction, and a reduction in collagen deposition time [25,26]. As already seen from a daily intake of olive oil, several scientific studies have reported that even olive oil creams applied topically have been proven to be effective for the treatment of patients with chronic wounds, having the ability to reduce the size, depth, and wound edges, along with associated pain, thanks to the antibacterial and anti-inflammatory effects of polyphenols [27,30]. In addition, it has been shown that HT may be a potential alternative therapeutic agent for the treatment of several autoimmune and inflammatory disorders. Indeed, in rheumatoid arthritis, it has the advantage of mitigating the systemic adverse effects of hydrocortisone [33]. Moreover, it displays anti-inflammatory and antioxidant benefits in the treatment of atopic dermatitis and psoriasis [35]. Despite its already known use in cosmetic formulations for antioxidant activities, the overall therapeutic potential of HT for the treatment of dermatological diseases has yet to be studied in detail, both in regard its beneficial activities and the molecular mechanisms implied.

Therefore, in our study, the effect of HT on human keratinocytes as an in vitro model was investigated in order to identify the dermatological properties of the molecule and provide solid biological bases for its transfer to the cosmetic or medical setting. 

We assessed HT activity in wound healing phases that are not well-defined. The wound is a pathological condition with the destruction of the physiological microstructure and a loss of tissue function by a chemical, thermal, or physical injury [49,50]. The wound healing process is mediated by the activation of local and systemic cells that cooperate to restore skin integrity [51]. It is distinguished by three different phases: inflammatory, proliferative of reepithelization, and remodeling [52].

The first inflammatory phase is characterized by vasodilation, followed by neutrophils infiltration, monocyte activation, and macrophage penetration into the wound site [53]. A non-physiological inflammatory state, too long, can stop the process and block the transition to the second phase. Indeed, excessive levels of inflammatory cytokines such as tumor necrosis factor alpha (TNF-α) or interleukin 6 (IL-6), IL-8, and IL-1β are implicated in a wound healing delay [54]. In this first phase, HT acts as a powerful anti-inflammatory molecule that relieves the inflammatory state favoring the wound healing progression [50].

On the other hand, little is known about HT activity in the second phase of the wound healing process. Cell proliferation and migration are critical cellular events in the second phase. Fibroblasts, keratinocytes, and endothelial cells are the three main actors involved in the human skin regeneration. First, fibroblasts migrate to the wound site and degrade the temporary fibrin matrix laid by the inflammatory phase cells [51]. Then, fibroblasts proliferate to form the granulation tissue and the re-epithelialization of the wound by the epithelial cells takes place, which migrate to the new matrix and proliferate [55]. At the same time, during the second phase, a new blood vessel is formed by the endothelial cells [56]. An increase in the proliferation and migration rate of keratinocytes undoubtedly leads to fast wound healing. In this wound healing second phase, our data are relevant, indeed, we show how HT has proliferative and migratory properties on keratinocytes, which undoubtedly represent the predominant cell type in the epidermis and have a fundamental role for the process of complete healing through re-epithelization and dynamic skin renewal [57].

Once the lack of HT cytotoxicity was confirmed even in the keratinocyte cell line, we found for the first time that concentrations in a low micromolar range (5–10 μM) induce DNA synthesis, stimulating cell proliferation. Our first result indicates that HT induces keratinocyte proliferation by increasing the protein expression of cyclin-dependent kinase, mainly CDK2 and CDK6, key components of the cell cycle mechanism for the transition from the G1 to S phase [37]. It is important to note that even if we used HaCaT cells, an immortalized cell line, as the model, basal keratinocytes physiologically maintain the ability to proliferate and only most terminally differentiated keratinocytes of the suprabasal layer are in a quiescent state, but can proliferate under appropriate stimuli [40,55].

In addition to cell proliferation, an adequate cell migration is also required for the second phase of wound healing for minor, superficial, and basic skin lesions, but also for more complicated pathological states such as ulcers or pressure sores [38,39].

We investigated if HT treatment could also influence the cell migration process. Several signaling events have been described as involved in the regulation of cell migration, extension, and contraction of the cytoskeleton, and in our cell model, HT induced an increase in the migration rate supported by the activation of MMP-9, ERK, PI3 Kinase, Akt, Rac1, Ras, and RhoA important factors for stimulating cell migration and normal tissue remodeling [40,41,42,43,58]. Successful wound healing requires movement of keratinocytes through the extracellular matrix. This process is facilitated by the activation of the gelatinolytic activity of metalloproteases as MMP-9, which are nevertheless also involved in the stimulation of cell proliferation, downstream cytokines, and growth factors stimulated signaling pathways that converge on Ras and ERK activation. Our results suggest that HT could show beneficial effects in wound healing not only by increasing keratinocyte proliferation, but also inducing cell migration. 

Oxidative stress is considered as a condition of imbalance in the reactive oxygen species (ROS) levels and the ability of biological systems to repair the resulting damage. Oxidative stress plays a crucial role in the onset of various diseases including allergic and inflammatory skin diseases such as atopic dermatitis, urticaria, and psoriasis [59,60] as well as in skin aging, and if it persists, it has been reported to inhibit cellular functions such as proliferation and migration, necessary for physiological wound healing [59].

Therefore, the regulation of skin ROS is essential for the maintenance of healthy skin homeostasis [60], but also for allowing a suitable wound healing [50]. We demonstrate the role of HT on the protection of cells by H_2_O_2_-induced DNA-damage and subsequent cell death. Our results confirm what is already reported in the scientific literature for the antioxidant activity of HT [36], but we demonstrated that this property is already evident at lower concentrations than previously reported (5 and 10 µM). Furthermore, as recent studies have revealed that HT has a very short half-life and the time required for its complete elimination is approximately 6 h [18,25], we believe that the effect shown by HT-treatment at 24 h (18 h + 6 h with H_2_O_2_) is not only due to the chemical effect of HT, but is attributable to the cellular response stimulated by HT. A rebalancing of the oxidative state is undoubtedly useful in reducing the destruction of the extracellular matrix (ECM) that provides nutrition for the microbes [61]. This scavenger activity of HT is added to the molecule’s ability to inhibit bacterial growth, as described in recent studies [50,62].

Furthermore, the second phase of the wound healing process includes a simultaneous angiogenesis necessary to restore the interrupted microcirculation necessary to support, with the supply of nutrients, the correct proliferation of the surrounding cells [56]. We and others have recently reported the proangiogenic activity of HT through the induction of cell proliferation and migration of vascular endothelial cells [63,64,65]. We observed that low concentrations of HT (1–5 µM), generally achieved after the ingestion of olive oil, are able to promote angiogenesis, inducing an increase in the expression of the vascular endothelial growth factor receptor (VEGF-R2) and activating the PI3K-Akt-eNOS protein pathway, recognized for its central role in angiogenesis [65]. In this context, it is conceivable that, beyond directly acting on keratinocytes and endothelial cells, HT could influence their reciprocal cross-talk in the multi-player milieu of wound healing, which depends on the integration of different signals and biological processes.

To date, nothing is known about the specific activity of HT in the last phase of the wound healing process, therefore further studies will be necessary in order to evaluate its potential in tissue remodeling and in maintaining balance between degradation and ECM synthesis. It will be necessary to investigate if HT also has a direct activity on fibroblasts and on keratinocyte–fibroblast interactions fundamental for the wound healing process. Moreover, it is important to determine if HT shows activity in the remodeling of the dermal structure during the formation of granulation tissue and if it plays a functional role in the transition from the fibroblast to the myofibroblast for the subsequent collagen deposition.

Our results allowed for a better understanding of HT beneficial activity in dermatological alterations using an in vitro model of keratinocyte. In addition to its anti-inflammatory and antioxidant activities, we provide, for the first time, evidence of increased cell proliferation and migration by identifying the proteins involved and the possible molecular mechanisms at the basis of these beneficial properties. 

In addition, the results obtained have identified the best concentrations for its biological activities and have paved the way for the development of highly effective HT-based cosmeceuticals.

Further studies will undoubtedly be needed in order to design formulations that contain effective concentrations of HT and allow for a continuous presence of its active form after topical application as well as a proper permeation of all skin layers.

## 4. Materials and Methods 

### 4.1. Chemicals and Materials

HT was purchased from Sigma-Aldrich Inc. (St Luis, MO, USA), solubilized in dimethyl sulfoxide (DMSO) (<0.001% in our assays). For western blot analysis, the following antibodies were used: mouse monoclonal antihuman CDK6, rabbit monoclonal antihuman CDK2, mouse monoclonal antihuman α-Tubulin, mouse monoclonal antihuman cyclin D3, rabbit monoclonal antihuman CD1, rabbit monoclonal anti-human phospho-ATM, rabbit monoclonal anti-human phospho-Chk1, rabbit monoclonal anti-human phospho-Chk2, rabbit anti-human phospho-p53, rabbit monoclonal anti-human phospho-p44/42 MAPK (p-Erk1/2; Thr202/Tyr204), rabbit monoclonal anti-human p44/42 MAPK (Erk1/2), rabbit monoclonal anti-human Phospho-Akt (p-Akt; Ser473), rabbit monoclonal anti-human Akt, rabbit monoclonal PI3-Kinase, rabbit monoclonal anti-human p53 (Cell Signaling Technology, Danvers, MA, USA). Mouse monoclonal MMP9, mouse monoclonal Rac1, rabbit polyclonal anti-human RhoA, rabbit monoclonal anti-human Ras, and rabbit polyclonal anti-human β-Actin were purchased from Abcam (Cambridge, UK). Secondary HRP-linked goat anti-mouse or goat anti-rabbit IgG were obtained from Cell Signaling Technology (Danvers, MA, USA). 

### 4.2. Cells

Human immortalized keratinocytes (HaCaT) were grown in Dulbecco’s modified Eagle’s medium (DMEM, GIBCO, Grand Island, NY, USA) and supplemented as described in detail elsewhere [66]. Keratinocyte cell cultures were maintained at 37 °C in a humidified 5% CO_2_ atmosphere.

### 4.3. MTT Assay for Cell Viability Determination

HaCaT cells (6 × 10^3^/well) were cultured for 24 h into 96-well plates before the addition of HT at the indicated concentrations and were cultured for an additional 24–48 h at 37 °C. To examine cell viability, we employed the reduction of the MTT (3-(4, 5-dimethylthiazolyl-2)-2, 5-diphenyltetrazolium bromide) tetrazolium salts assay, as described in detail elsewhere [67]. All experiments were performed in triplicate, and the relative cell viability was expressed as percentage in comparison with the untreated control cells.

### 4.4. BrdU Assay for Cell Proliferation Determination

HaCaT cells (6 × 10^3^/well) were cultured for 24 h into 96-well plates before the addition of HT at the indicated concentrations and were cultured for an additional 24–48 h at 37 °C. BrdU incorporation into DNA (BrdU colorimetric assay kit; Roche Applied Science, South San Francisco, CA, USA) was used to measure cell proliferation, determined by an ELISA (enzyme-linked immunosorbent assay) plate reader (ThermoScientific, Waltham, MA, USA) at 450 nm as described in detail elsewhere [68]. All experiments were performed in triplicate, and the relative cell growth was expressed as a percentage in comparison with the untreated control cells.

### 4.5. Scratch Wound Healing Assay

For cell migration evaluation, HaCaT cells were plated in 6-well plates at a density of 5 × 10^3^ cells/well. When the confluent cells formed a homogeneous carpet and a vertical wound in the wells using a 200 µL tip was performed, culture medium containing HT at 1, 5, and 10 µM or the vehicle alone was added to the wells, after two washes to eliminate the cells detached. The wound area was recorded immediately and after 18 h through microscope analysis, as previously described [69] and quantified by Wimasis Image Analysis software (Onimagin Technologies Spa, Cordoba, Spain).

### 4.6. Apoptosis Analysis

Quantitative assessment of apoptosis of HaCaT cells was analyzed by anti-human annexin V (BioLegend, San Diego, CA, USA) using propidium iodide solution (PI) staining. Briefly, cells grown in p60 tissue culture plates for 24 h with HT at 1, 5, and 10 µM, H_2_O_2_ or combined as indicated, were harvested with trypsin, washed in phosphate buffer saline (PBS), and subjected to apoptosis determination by the procedure described in detail elsewhere [70].

### 4.7. Western Blot Analysis

Cells were grown in p100 tissue culture plates at a density of 2 × 10^4^ cells/cm^2^ for 24 h. Cells were then incubated with HT, H_2_O_2_ (for 6 h), or their combination (HT for 18 h and H_2_O_2_ for an additional 6 h) at the indicated concentrations. Subsequently, cells were washed with PBS, harvested, and lysed in ice-cold radioimmunoprecipitation assay (RIPA) lysis buffer (50 mM Tris-HCl, 150 mM NaCl, 0.5% Triton X-100, 0.5% deoxycholic acid, 10 mg/mL leupeptin, 2 mM phenylmethylsulfonyl fluoride, and 10 mg/mL aprotinin) and then assayed for Western Blot (WB) by the procedure described in detail elsewhere [71].

### 4.8. Statistical Analysis

Statistical analysis was performed by GraphPad prism 6.0 software for Windows (GraphPad software, San Diego, CA, USA). For each type of assay, data obtained from multiple experiments were calculated as mean ± (SD) and analyzed for statistical significance using the 2-tailed Student t-test for independent groups, or 2-way ANOVA followed by Tukey post-hoc correction for multiple comparisons. *P* values < 0.05 were considered significant. * *p* < 0.05, ** *p* < 0.01, *** *p* < 0.001, and **** *p* < 0.0001.

## 5. Conclusions

In conclusion, the novelty of our in vitro study is that we provide the first demonstration of how specific HT concentrations of 5 and 10 µM are able to promote keratinocyte cell migration and proliferation in vitro. HT has the potential to enhance re-epithelialization, at the same time, protecting the skin from the action of free radicals thanks to its anti-oxidant properties, also confirmed in our keratinocyte model. Our data highlight the great pharmaceutical potential for HT through multi-targeted actions, paving the way for possible cosmeceutical applications. Additional in vivo and clinical studies are required to develop and validate novel cosmeceuticals and pharmacological formulations based on HT.

## Data Availability

The data presented in this study are available on request from the corresponding author.
